# Symptoms of Psychological Stress and Sickness Absence Among Healthcare Workers During a Persistent Crisis

**DOI:** 10.1111/sjop.13127

**Published:** 2025-05-26

**Authors:** Sophia Appelbom, Anna Finnes, Rikard K. Wicksell, Aleksandra Bujacz

**Affiliations:** ^1^ Department of Learning, Informatics, Management and Ethics Karolinska Institutet Stockholm Sweden; ^2^ Department of Clinical Neuroscience Karolinska Institutet Stockholm Sweden; ^3^ Academic Primary Health Care Centre Region Stockholm Stockholm Sweden; ^4^ Pain Clinic Capio St Göran Hospital Stockholm Sweden

**Keywords:** COVID‐19 pandemic, crisis, healthcare workers, latent profile analysis, psychological stress, sickness absence

## Abstract

Elevated psychological stress reactions among healthcare workers during the COVID‐19 pandemic necessitated a need to better understand their possible impact on sickness absence (SA). The study aimed to describe the relation between SA related to mental health problems and symptoms of psychological stress among healthcare workers during the COVID‐19 pandemic. We further aimed to identify whether latent profiles of psychological stress reactions exist within the same population. In this observational registry‐based study, survey data between May 2020 and March 2021 and SA register data between May 2019 and February 2023 were collected from 1245 Swedish healthcare workers. Differences between symptoms of burnout, depression, anxiety, PTSD, sleep disturbance, lack of detachment, and lack of recovery among groups with no, few (< 90), or many (≥ 90) SA days were analyzed with Kruskal‐Wallis tests. Interrelations between symptoms of psychological stress were identified using latent profile analysis. Compared to healthy participants, participants with SA days (in total 6.3%) reported more severe symptoms of psychological stress, were younger, and more likely to work as assistant nurses. Furthermore, they displayed a higher degree of previous SA (prior to the pandemic). No statistically significant differences between groups with few (< 90) and many (≥ 90) days of SA in symptoms were noted. Four latent profiles of psychological stress were identified, but they differed only in the level of experienced symptoms. We conclude that different symptoms of psychological stress are highly interrelated among healthcare workers during a crisis. Although many healthcare workers may experience elevated symptoms in relation to the crisis, it will not necessarily lead to serious mental health problems requiring SA.

**Summary**

- Few studies have investigated the relation between symptoms of psychological stress and mental health related Sickness Absence (SA) among healthcare workers during the COVID‐19 pandemic.
- The study aimed to (a) describe the relation between SA related to mental health problems and symptoms of psychological stress, and (b) identify whether latent profiles of psychological stress reactions exist among healthcare workers during the COVID‐19 pandemic.
- The results showed that healthcare workers with a mental health related SA had higher ratings on symptoms of psychological stress than healthcare workers with no days of SA.
- The latent profiles of psychological stress symptoms varied only in level of experienced symptoms, indicating that different symptoms of psychological stress are highly related among healthcare workers during a crisis.
- Elevated symptoms of psychological stress will not necessarily lead to SA, more research is needed on how other factors may contribute to mental health related SA among healthcare workers during a crisis.

## 1 Introduction

Globally, the prevalence of mental health disorders among the working population has grown over recent years (WHO 2022b). Sickness absence (SA) related to mental health problems has long rehabilitation times (Demou et al. 2018) and costs are large for both healthcare and society (Dobson et al. 2019; Soni 1996). SA episodes related to mental health problems have been a large concern within the healthcare sector during recent decades (Hansson et al. 2006; Michie and Williams 2003). In Sweden, mental health problems are the most common reason behind sickness benefits related to long‐term SA (Swedish Social Insurance Agency 2023) and healthcare workers exhibit elevated rates of mental health related SA compared to other occupations within comparable sectors and educational levels (AFA Försäkring 2023). Between different medical occupations, SA is proportionally more common among nurses and assistant nurses than among physicians (AFA Försäkring 2020).

A complex pattern of organizational, social, and individual factors often influences the risk of SA related to mental health problems (de Vries et al. 2018). Previous history of SA and mental health disorders increases the risk of long‐term SA later in life (de Vries et al. 2018; Gustafsson and Marklund 2011); women are overrepresented compared to men (de Vries et al. 2018; Svedberg et al. 2018), and social factors such as work‐home conflicts and children living at home also contribute to increased risk (Svedberg et al. 2018). Importantly, SA is more common in professions where the work environment is characterized by high job demands, high workload, and low support (de Vries et al. 2018; Duchaine et al. 2020; Niedhammer et al. 2012; Svedberg et al. 2018). Besides social and organizational factors, high ratings on symptoms of psychological stress are a major predictor of SA related to mental health problems (Duijts et al. 2007; Hansson et al. 2006; Svedberg et al. 2018).

Still, high symptoms can be experienced during shorter periods without leading to a clinical diagnosis, either because the exposure to a stressor ceases or through good coping strategies (De Jonge and Dorman 2017). Psychological recovery outside of work, that is, unwinding and switching off from work, is an important buffering factor that helps individuals cope with high stress levels (Sonnentag and Fritz 2015) and prevents future sickness absence (Boschman et al. 2017).

During the first year of the COVID‐19 pandemic, psychological stress reactions, such as symptoms of burnout, depression, anxiety, posttraumatic stress disorder, and sleep disturbance, increased among healthcare workers (Luo et al. 2020; Pappa et al. 2020; Salari et al. 2020; Salazar de Pablo et al. 2020). In Sweden, healthcare workers who reported early signs of both burnout and depressive symptoms were also more likely to report prolonged symptoms during the first 18 months of the pandemic (Appelbom et al. 2024). While there are many studies investigating mental health aspects among healthcare workers during the COVID‐19 pandemic, studies connecting symptoms of psychological stress and mental health‐related SA are scarce, and they have not used register‐based SA data (Dolić et al. 2022; Edge et al. 2022; Van Der Plaat et al. 2021). Due to the elevated psychological stress reactions among healthcare workers during the COVID‐19 pandemic, there is a need to predict and prevent the development of SA in connection to future crises (Gohar et al. 2020).

There is also a complexity of comorbidity and overlap among symptoms of different types of psychiatric conditions (Bianchi et al. 2015; Orosz et al. 2017; Roca et al. 2009). These kinds of symptom interactions are highlighted in studies applying person‐centered analysis (e.g., latent profile or class analysis), which commonly identify one large group of participants with low symptom levels and smaller groups that have severe symptoms with large comorbidity among symptoms of depression, anxiety, and posttraumatic stress disorder (Kessler et al. 2005; Vaidyanathan et al. 2011). Likewise, studies identifying burnout profiles have found that participants with high burnout symptoms also had higher depression and anxiety levels as well as more sick leave (Boulier et al. 2021; Schult et al. 2018). Consequently, taking the variations in how different symptoms interrelate into account may increase the understanding of psychological stress among healthcare workers both with and without SA related to mental health problems during the COVID‐19 pandemic.

## 2 The Present Study

In this observational registry‐based study, we investigated how symptoms of psychological stress are interrelated and whether they are associated with different rates of SA related to mental health problems among healthcare workers during the persistent healthcare crisis caused by the COVID‐19 pandemic. Specifically, we analyzed how symptoms of psychological stress interrelate both in terms of coexistence and severity within this population. Furthermore, we aimed to describe differences in symptoms of psychological stress between groups of healthcare workers with no, few (< 90), and many (≥ 90) days of mental health‐related SA.

## 3 Materials & Methods

### 3.1 Participants and Procedure

Participants in a research project investigating the psychosocial work environment and mental health among healthcare workers in Sweden during the COVID‐19 pandemic and who consented to the extraction of data on sickness absence were included in the study. Recruited participants were employees from hospitals and primary care. The surveys were administered between May 2020 and March 2021.

A total of 1431 survey participants consented to the extraction of data on registered sickness absence from the Swedish Social Insurance Agency and were therefore eligible for the present study. Out of the total sample, 1245 participants had scores on each psychological stress measurement and were therefore included in the analytic sample. See the Supporting Information S1 for details on the recruitment process, sub‐cohorts, data collection, and missing data concerning dropouts.

### 3.2 Measures

#### 3.2.1 Work Characteristics

COVID‐19 specific aspects of the work environment were measured with two items: Frontline work and Changed work tasks. *Frontline work* was indicated by participants stating whether they had treated COVID‐19 patients daily or several times during the last week. The variable *Changed work tasks* measured whether participants had received changed work tasks due to the COVID‐19 pandemic.

#### 3.2.2 Sickness Absence

Data on ICD‐10 diagnosis and SA from 12 months prior and up to 24 months after study inclusion was collected from the Swedish Social Insurance Agency in February 2023. Due to time lag in the reporting of cases, for 835 participants the upper time limit was shorter than 24 months (between 20 and 23 months). Three SA variables were used in the study.

##### 3.2.2.1 SA Days During the COVID‐19 Pandemic

The number of SA days during the COVID‐19 pandemic was calculated as net SA days, that is, full‐day equivalents of part‐time SA since the start of the pandemic. An employee who is on leave 50% for 10 days would therefore be counted as 5 net‐days of SA. The same applies to a part‐time employee who works 50% but is on leave full‐time. The start of the pandemic referred to March 11, 2020, when WHO declared that the COVID‐19 outbreak had reached the state of a pandemic (WHO 2020). For each participant, the number of net SA days due to a psychiatric ICD‐10 diagnosis maintained during this period was categorized into a 3‐level variable consisting of no SA days (0), few (< 90) SA days (1), or many (≥ 90) SA days (2). The cut‐off was based on the definition of long‐term SA as episodes of 90 days that is commonly used in Sweden (AFA Försäkring 2020).

##### 3.2.2.2 History of Mental Health SA

Pre‐pandemic history of SA related to mental health problems was measured as episodes of SA motivated by a psychiatric ICD‐10 diagnosis with a starting point before March 11, 2020. For the analysis, this variable was categorized as no previous SA episodes (0) or at least one previous SA episode (1). Depending on when participants entered the study, the period of potential pre‐pandemic SA episodes varied between 0 and 10 months.

##### 3.2.2.3 Non‐Psychiatric SA

Episodes of sickness absence motivated by non‐psychiatric ICD‐10 diagnoses were categorized as no episode of non‐psychiatric SA (0) or at least one other existing episode of non‐psychiatric SA (1). This variable did not differentiate between episodes before or after the start of the COVID‐19 pandemic.

#### 3.2.3 Symptoms of Psychological Stress

Seven different symptoms of psychological stress were measured in the study: burnout, depressive symptoms, anxiety, post‐traumatic stress, sleep disturbance, lack of detachment, and lack of recovery.

*Burnout* symptoms were measured using a 6‐item version of the Shirom‐Melamed Burnout Questionnaire (SMBQ‐6). SMBQ‐6 measures cognitive weariness and both emotional and physical exhaustion. SMBQ is a validated screening tool for detecting clinical burnout symptoms (Almén and Jansson 2021). Participants rated the 6 items on a 7‐point scale, with scale anchors *almost never* (1) and *almost always* (7). The scale mean was 3.16 (SD = 1.55, *α* = 0.921, *ω* = 0.923).

*Depressive symptoms* were measured with the 2‐item version of the Patient Health Questionnaire (PHQ‐2). The 2‐item version of PHQ measures the frequency of symptoms of anhedonia and low mood within the past 2 weeks (Gilbody et al. 2007). Participants rated items on a 4‐point scale, ranging from not at all (1), several days (2), more than half of the days (3), to nearly every day (4). The scale mean was 1.77 (SD = 0.87, Spearman's rho = 0.67).

*Anxiety* was measured with the Generalized Anxiety Disorder‐7 questionnaire (GAD‐7). GAD‐7 is a seven‐item long questionnaire developed to both screen for and measure the severity of Generalized Anxiety Disorder (Bischoff et al. 2020; Spitzer et al. 2006). Participants were asked how often they had been bothered by seven different symptoms of anxiety within the past 2 weeks. Items were rated on a 4‐point scale, ranging from not at all (1), several days (2), more than half of the days (3), to nearly every day (4). The scale mean was 1.57 (SD = 0.61, *α* = 0.894, *ω* = 0.897).

*Symptoms of posttraumatic stress disorder* were measured using three items from the Posttraumatic Stress Disorder Checklist (PCL‐5). PCL‐5 measures post‐traumatic stress symptoms as they are formulated in DSM‐5 (Blevins et al. 2015). The items measured repeated and disturbing memories of the stressful experience, avoidance related to the stressful experience, and the tendency to be hyperalert. To measure post‐traumatic stress in relation to events at work, participants were asked how much they had been bothered by each symptom in relation to their work situation. Items were rated on a 5‐point scale, ranging from not at all (1), a little bit (2), moderately (3), quite a bit (4), to very much (5). The scale mean was 1.94 (SD = 0.98, Spearman's *r* = 0.671).

*Sleep disturbance* was measured with one item. Participants were asked how they had slept during the past week. Answers were rated on a seven‐point scale ranging from very good (1), good (2), fairly good (3), neither good nor bad (4), fairly bad (5), bad (6), to very bad (7). The item mean was 3.43 (SD = 1.59).

*Lack of detachment* was measured with a single item where participants were asked to indicate to what extent they had been able to stop thinking about work during their leisure time within the last week. Answers were rated on a 4‐point scale ranging from very often or always (1), fairly often (2), fairly seldom (3), to very seldom or never (4). The item mean was 2.49 (SD = 1.01).

*Lack of recovery* was measured with a single item. Participants were asked how often they had engaged in activities promoting their wellbeing (e.g., exercise or other hobbies) within the last week. The item was rated on a 4‐point scale ranging from daily (1), several times (2), at some point (3), to never (4). The item mean was 2.42 (SD = 0.92).

### 3.3 Statistical Analyses

Demographical differences between the three SA groups (i.e., no SA days, short‐term SA, and long‐term SA) were compared using Pearson's chi‐square test for count data on categorical variables. A post hoc analysis was performed by inspecting standardized residuals, where residuals with absolute values > 1.96 indicate a statistically significant deviation from the expected count on a *p* < 0.05 level (Agresti 2002). Age differences were tested using one‐way Kruskal‐Wallis tests, with Dunn's Test using Bonferroni adjusted *p*‐values as a post hoc test. When comparing gender distributions, only categories of women and men were included.

To describe differences in symptoms of psychological stress between groups of healthcare workers with no, few (< 90), and many (≥ 90) days of mental health related SA, differences in symptoms of psychological stress were tested using one‐way Kruskal‐Wallis tests with Dunn's Test using Bonferroni adjusted *p*‐values as a post hoc test. This non‐parametric test was used due to the unequal group sizes of the SA groups. Effect size was calculated using epsilon squared estimates, with values close to 0 indicating no relationship and values close to 1 indicating a perfect relationship (Tomczak and Tomczak 2014). To ease the interpretation of the diverse symptoms, grand mean centered symptoms were then plotted for each SA group separately.

To analyze how symptoms of psychological stress interrelate in terms of coexistence and severity, a latent profile analysis (LPA) was performed to identify profiles of psychological stress symptoms. Latent profile analysis is a person‐centered approach that assumes heterogeneity within the sample. Participants can therefore be categorized into profiles based on indicators of similar characteristics (Morin et al. 2020). The analysis was conducted following the guidelines proposed by van Lissa et al. (2023). We used the measures of psychological stress symptoms as indicators, centered around a variable's grand mean. When centered, positive and negative values indicate scores above or below the variable mean. Models with one to eight profiles were tested. The final profile solution was chosen based on model fit indicators (BIC, AIC, and SABIC) and our theoretical expectation to find latent profiles with different combinations and severity of psychological stress symptoms (Nylund‐Gibson and Choi 2018).

All analyses were conducted using R version 4.3.0 (R Core Team 2023). The code is available in the Supporting Information S1. The following packages were used: rcompanion for chi‐square tests, post hoc tests using standardized residuals, and calculation of effect sizes (Magnifico 2024); FSA for post hoc tests using Dunn's test (Ogle et al. 2023); and tidySEM for the latent profile analysis (van Lissa 2023).

## 4 Results

Characteristics of the analytic sample are presented in Table 1.

**TABLE 1:** Demographic characteristics of the analytic sample.

Characteristic	*n*	%
Gender		
Men	274	22.0
Women	968	77.9
Other	1	0.1
Occupation		
Assistant nurse	186	15.0
Nurse	451	36.5
Physician	207	16.7
Other professions	393	31.8
Children at home	602	48.7
Tenure		
0–1 years	35	2.9
2–5 years	213	17.7
6–10 years	185	15.4
11+ years	771	64.0
Sought help for mental health		
Never	952	76.8
Within last 3 months	70	5.6
> 3 months prior	218	17.6
Frontline work	426	36.1
Changed work tasks	551	44.5
SA COVID‐19 period		
No days	1166	93.7
< 90 days	44	3.5
≥ 90 SA days	35	2.8
History of mental health SA	27	2.2
Non‐psychiatric SA	290	23.3

*Note: N* = 1245. The mean age of participants was 46.4 years (SD = 11.78). Frontline work was defined as having treated COVID‐19 patients several times or every day within the last week.

### 4.1 Demographic Differences Between SA Groups

Demographic characteristics of the analytic sample are presented in Table 1. Out of the 1245 participants, the majority of the sample 93.7% (*n* = 1166) had no records of SA days related to mental health problems, while the remaining 6.3% (*n* = 79) had SA related to mental health problems at some point from the start of the COVID‐19 pandemic. In terms of number of SA days, 3.5% (*n* = 44) of the participants belonged to the few (< 90) SA days group, and 2.8% (*n* = 35) to the many (≥ 90) SA days group.

Differences in demographic characteristics between the SA groups are presented in Table 2. Post hoc tests using Dunn's test showed that participants with many SA days were younger compared to the no SA group (Δ*M* = 7.53, *z* = 3.676, *p* < 0.001). Standardized residuals indicated that there were more assistant nurses in both the few and many SA days groups compared to the no SA group (20.5% and 25.7% compared to 14.5%), while physicians were overrepresented in the no SA group (17.8% compared to 2.3% and 0%). In the few and many SA days groups, it was also more common to have sought previous treatment for mental health issues both > 3 months (38.6% and 37.1% respectively, compared to 16.2% in the no SA group) and < 3 months prior to inclusion (20.5% and 25.1% respectively vs. 4.5% in the no SA group), see Table 2.

**TABLE 2:** Demographic characteristics of the SA groups.

Characteristic	No SA days		Few (< 90); SA days		Many (≥ 90); SA days		*H*/*χ* ^2^	df	*p*
	*M*/*n*	SD/%	*M*/*n*	SD/%	*M*/*n*	SD/%			
Age	46.68^a^	11.79	43.07	10.70	39.15^a^	9.98	16.97	2	< 0.001
Gender									
Men	263	22.6	7	16.3	4	11.4	3.330	2	0.189
Women	901	77.4	36	83.7	31	88.6			
Occupation									
Other	367	31.7	14	31.8	12	34.3	17.087	6	0.009
Assistant nurses	168*	14.5	9	20.5	9	25.7			
Nurses	417	36.0	20	45.5	14	40.0			
Physicians	206*	17.8	1*	2.3	0*	0.0			
Children	563	48.6	20	45.5	19	55.9	0.894	2	0.640
Tenure									
0–1 years	33	2.9	2	4.8	0	0.0	10.783	6	0.095
2–5 years	192	17.0	10	23.8	11	31.4			
6–10 years	169	15.0	9	21.4	7	20.0			
> 10 years	733	65.0	21	50.0	17	48.6			
Treatment for mental health									
Not prior inclusion	921*	79.3	18*	40.9	13*	37.1	80.368	4	< 0.001
> 3 months prior inclusion	188*	16.2	17*	38.6	13*	37.1			
< 3 months prior inclusion	52*	4.5	9*	20.5	9*	25.7			
Frontline work	401	36.3	14	34.1	11	32.4	0.286	2	0.867
Changed tasks	522	45.1	18	40.9	11	31.4	2.806	2	0.246
History of mental health SA	21*	1.8	5*	11.4	1	2.9	18.355	2	< 0.001
Non‐psychiatric SA	259*	22.2	20*	45.5	11	31.4	14.153	2	< 0.001

*Note:* Percentages are summed up per SA group (column) within each variable. Means marked with analogous letter within the age row differ at the *p* = 0.05 level by Dunn's Test (with Bonferroni adjusted *p*‐values). Counts marked with asterisks have standardized residuals with absolute values > 1.96, which indicates a deviance on the *p* < 0.05 level from the expected count.

The SA groups also differed in terms of previous mental health SA. Standardized residuals indicated that there was an underrepresentation of those with a previous mental health SA in the no SA group (1.8%) and overrepresentation in the few SA days group (11.4%), but not in the many SA days group, with an overrepresentation in the few SA days group (45.5%) and fewer cases in the no SA group (22%). There were no statistically significant differences between the groups on gender distribution, number of children living at home, tenure, frontline work, and changes in work tasks due to the pandemic; see Table 2.

### 4.2 Differences in Symptoms of Psychological Stress Between SA Groups

Kruskal‐Wallis test showed statistically significant differences between SA groups in depressive symptoms, PTSD, anxiety, burnout, and lack of recovery. There were no significant differences between the three groups for either sleep disturbance or lack of detachment; see Table 3.

**TABLE 3:** Score means (SD) for the symptoms of psychological stress by SA groups.

Measure	No SA days		Few (< 90); SA days		Many (≥ 90); SA days		*H*(2)	*ε* ^2^	*p*
	*M*	SD	*M*	SD	*M*	SD			
Lack of detachment	2.48	1.01	2.68	1.01	2.80	0.93	4.979	0.004	0.082
Lack of recovery	2.40^a^	0.91	2.84^a^	0.94	2.51	0.95	9.928	0.008	0.006
Sleep disturbance	3.39	1.56	3.98	1.96	4.03	1.79	8.109	0.007	0.0174
Depressive symptoms	1.73^ab^	0.84	2.35^a^	1.01	2.24 ^b^	1.10	24.916	0.020	< 0.001
PTSD	1.90^ab^	0.95	2.48^a^	1.06	2.63^b^	1.25	26.804	0.022	< 0.001
Anxiety	1.54^ab^	0.59	2.02^a^	0.77	1.96^b^	0.77	34.811	0.028	< 0.001
Burnout	3.07^ab^	1.50	4.54^a^	1.50	4.44^b^	1.77	51.782	0.042	< 0.001

*Note:* Means marked with analogous letters within each row differ at the *p* = 0.05 level by Dunn's Test (with Bonferroni adjusted *p*‐values). *ε* ^2^ with values close to 0 indicates no relationship and values close to 1 indicate a perfect relationship.

**FIGURE 1:**
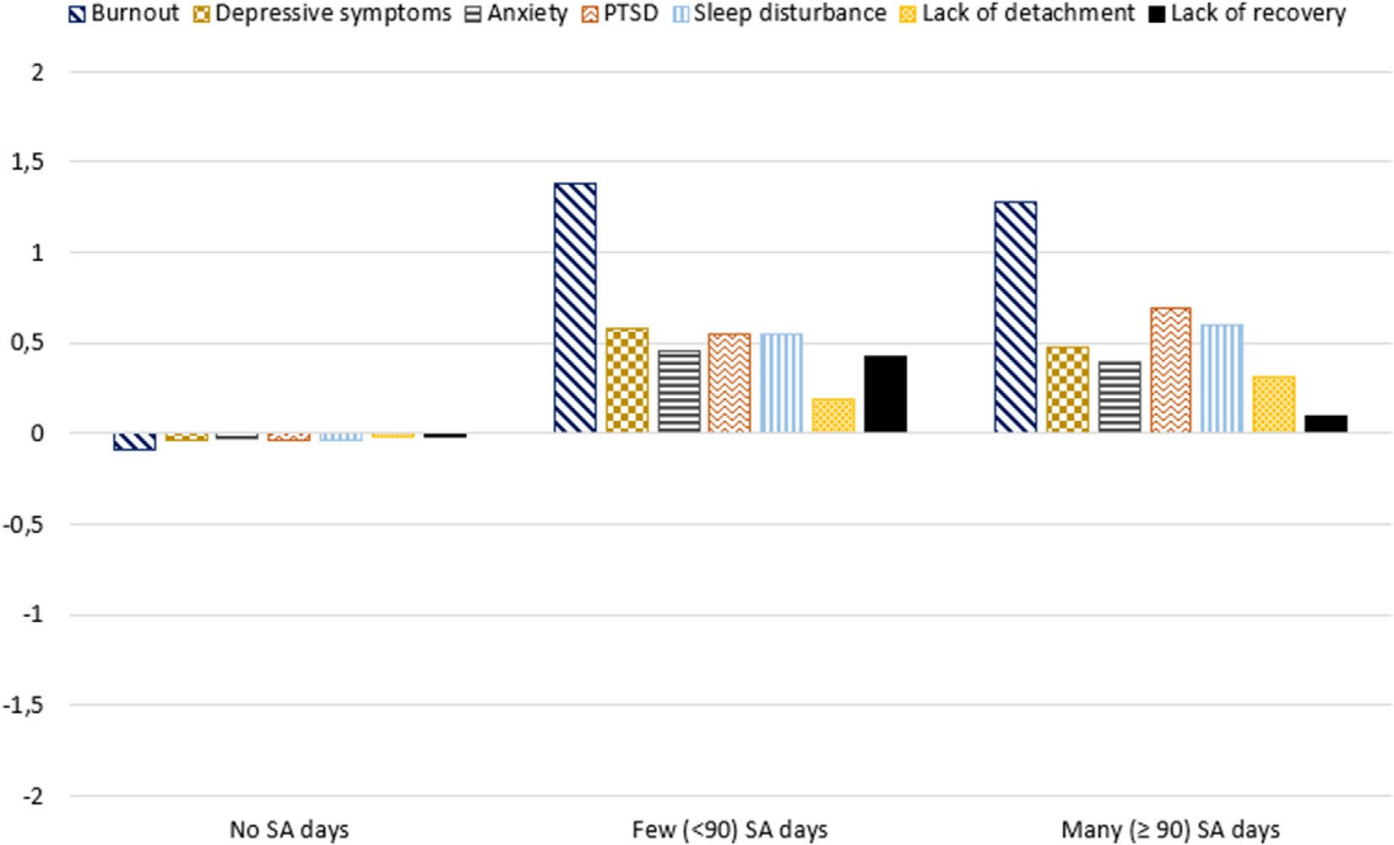
Mean values of grand mean centered symptoms of psychological stress for the SA groups.

Dunn's tests indicated that compared to the group with no SA days, the few SA days group had higher means on depressive symptoms (Δ*M* = 0.62, *z* = −4.25 *p* < 0.001), PTSD (Δ*M* = 0.59, *z* = −3.97, *p* = 0.0017), anxiety (Δ*M* = 0.48, *z* = −4.57 *p* < 0.001), burnout (Δ*M* = 1.47, *z* = −5.78, *p* < 0.001), and lack of recovery (Δ*M* = 0.44, *z* = −3.07, *p* = 0.006). Also, compared to the no SA days group, the many SA days group had higher means on depressive symptoms (Δ*M* = 0.51, *z* = −2.753, *p* = 0.018), PTSD (Δ*M* = 0.729, *z* = −3.46, *p* < 0.001), anxiety (Δ*M* = 0.43, *z* = −3.88, *p* < 0.001), and burnout (Δ*M* = 1.37, *z* = −4.47, *p* < 0.001), but not on lack of recovery. There were no significant differences indicated between the few and many SA days groups. See Table 3 for mean values, standard deviation, and significance test results for all symptom variables. Grand mean centered scores by SA groups are plotted in Figure 1.

### 4.3 Identifying Latent Profiles of Symptoms of Psychological Stress

**FIGURE 2:**
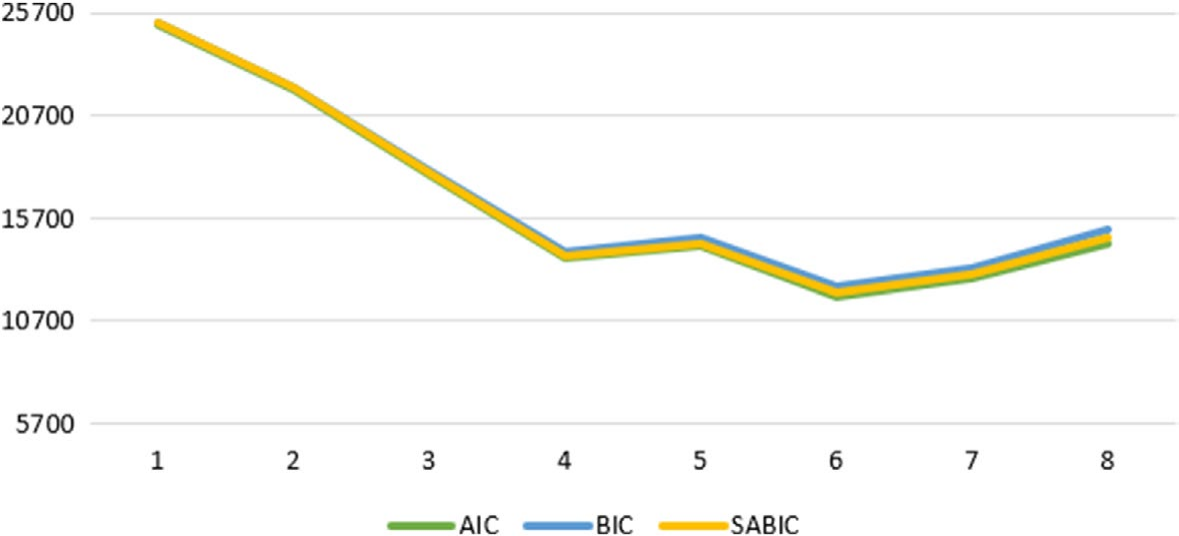
Elbow plot with fit statistics from the latent profile analysis.

When testing models with different numbers of profiles, the BIC, AIC, and SABIC values decreased for each model until the 4‐profile solution. For models 5–8, the fit was not consistent between models (both increased and decreased). When plotting the AIC, BIC, and SABIC values, the line started to level off at the 4‐profile solution (Figure 2). Due to weak evidence for the superiority of the models with more profiles, the model with four profiles was chosen to avoid interpreting possibly spurious profiles. Model fit statistics are presented in Table 4. Average posterior probabilities (AvePP) > 0.7 indicated well‐separated profiles for the four‐profile solution (Nylund‐Gibson and Choi 2018; van Lissa et al. 2023), see Table 5.

**TABLE 4:** Model fit statistics.

*k*	LL	#*p*	AIC	BIC	SABIC	Entropy	*n* min
1	−12,566	14	25,160.04	25,231.82	25,187.35	1	100.00
2	−10,943.9	29	21,945.87	22,094.55	22,002.44	0.917444	38.71
3	−8860.2	44	17,808.39	18,033.98	17,894.21	0.905294	29.00
4	−6816.62	59	**13,751.25**	**14,053.74**	**13,866.33**	0.928856	11.73
5	−7084.65	74	14,317.29	14,696.68	14,461.62	0.971000	10.28
6	−5842.2	89	11,862.39	12,318.69	12,035.98	0.971206	10.36
7	−6263.41	104	12,734.83	13,268.03	12,937.67	0.962931	3.45
8	−7118.21	119	14,474.41	15,084.51	14,706.52	0.900579	3.21

*Note: k* = number of latent profiles in the model; LL = model log likelihood; #*p* = number of parameters; *n* min = size of the smallest profile (%). *N* = 1245.

Abbreviations: AIC = Akaike information criterion, BIC = Bayesian information criterion, SABIC = sample size adjusted BIC.

**TABLE 5:** Average posterior probabilities by most likely profile pattern and profile size.

	Profile 1	Profile 2	Profile 3	Profile 4
Profile 1	**0.9941**	0.0000	0.0000	0.0059
Profile 2	0.0000	**0.9440**	0.0544	0.0016
Profile 3	0.0000	0.0735	**0.9264**	0.0000
Profile 4	0.0002	0.0008	0.0000	**0.9989**
Profile size *n* (%)	146 (11.73)	350 (28.11)	409 (32.85)	340 (27.31)

*Note:* Profile mean probabilities (columns) by profile assignment (rows). Profile size based on the most likely profile.

#### 4.3.1 Description of Profiles

**FIGURE 3:**
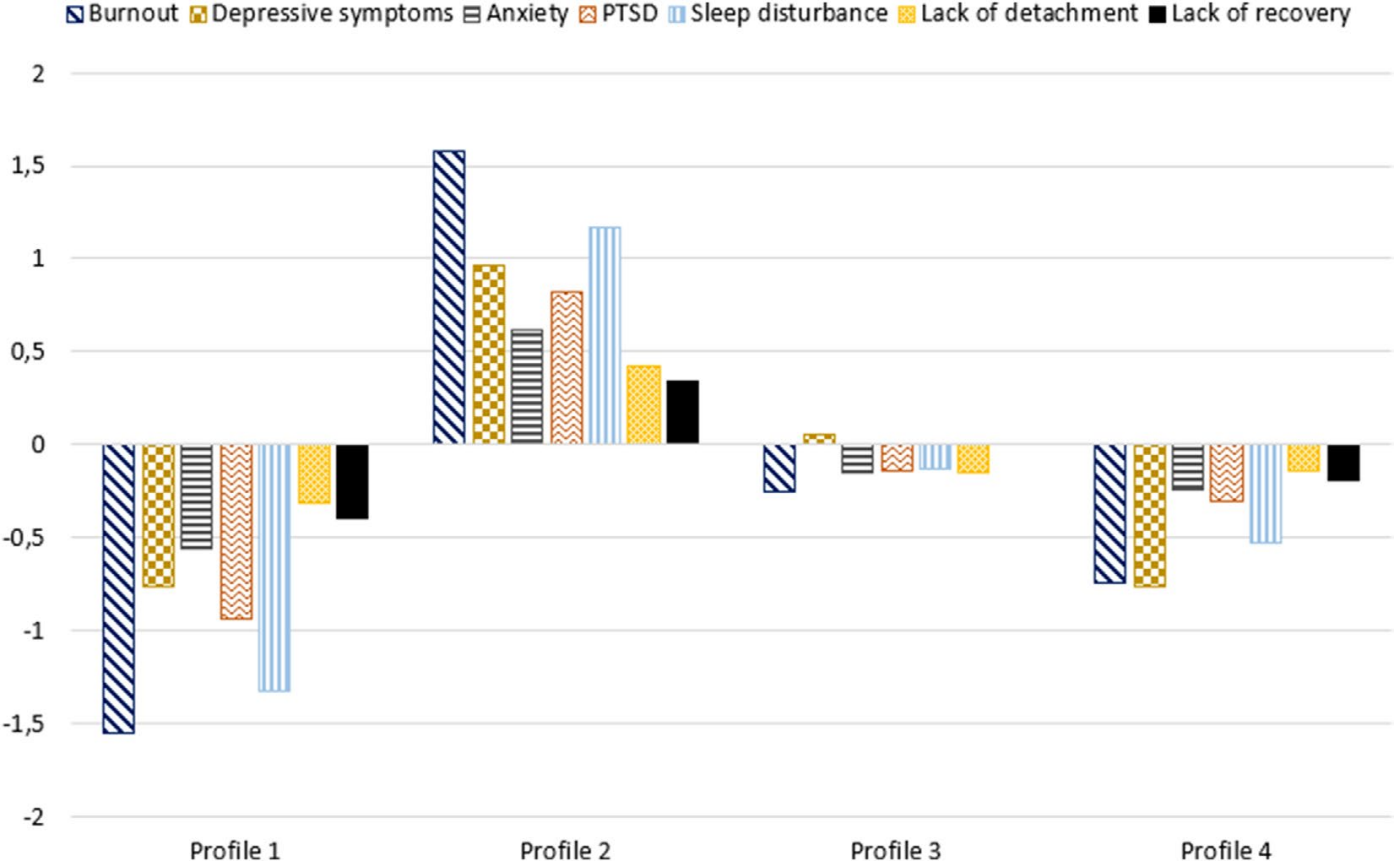
Final four‐profile solution. Grand mean centered scores of each indicator by profile.

All symptoms of psychological stress followed a similar pattern within the profiles and varied in severity between profiles. The profiles were categorized by low, moderate, or high levels of all symptoms. Healthcare workers included in Profile 1 accounted for 11.73% of the sample and had low scores, below the scale means, on all symptoms. Those in Profile 2 had the highest symptoms, above the scale mean across all indicators, and accounted for 28.11% of the sample. In these profiles, burnout and sleep disturbance differentiated more than one point from the scale means and the symptoms differed more than two points between the profiles. Profile 3 accounted for 32.85% of the sample and 27.31% of the participants were classified to Profile 4. Healthcare workers belonging to these profiles had scores either around or just below the scale means across all symptoms, with scores differentiating < 1 point from the mean. The final four‐profile solution with grand centered means is presented in Figure 3.

## 5 Discussion

In this study, we used self‐reported and register data to investigate profiles of psychological stress symptoms and SA related to mental health problems among healthcare workers during the first 2 years of the COVID‐19 pandemic. While the majority of participants did not have any registered SA, 6.3% had mental health‐related SA during the study period. In this group, more participants were younger, worked as assistant nurses, and had a previous history of SA due to both mental health and somatic diagnoses.

When investigating how various symptoms of psychological stress interrelate among healthcare workers during a crisis, we found that symptoms varied mainly in severity between the four latent profiles. These results indicate that the coexistence of different symptoms of psychological stress was common among healthcare workers during the COVID‐19 pandemic, in line with what is known about comorbidity among mental disorders (Kessler et al. 2005; Vaidyanathan et al. 2011). The results also align with prior research which posits that different symptoms of psychological stress often reoccur among psychiatric conditions (Bianchi et al. 2015). They may therefore be a good predictor of future mental health related sick leave (Hansson et al. 2006), but are less accurate in differentiating between different psychiatric conditions (Roca et al. 2009).

Comparing the symptom levels of the four profiles with the three SA groups (see Figures 1 and 3), participants from both the few and many SA days groups had a similar pattern of psychological stress symptoms as members of Profile 2. Hence, if an auxiliary analysis with a model testing the association between SA groups and profiles had been performed (Morin et al. 2020; van Lissa et al. 2023), membership in Profile 2 had likely been associated with days of SA (both < 90 and ≥ 90 days). Although healthcare workers in the no SA group had moderate levels of all symptoms, the variation in the level of symptoms between the four profiles indicates that there likely is a large variation in psychological stress reactions also in this group, ranging from low to high levels.

Participants with both few and many SA days had higher ratings on all symptoms of psychological stress, compared with the no SA group. This association between severe symptoms and SA indicates that the observed increase in psychological stress reactions among healthcare workers at the outbreak of the COVID‐19 pandemic (Luo et al. 2020; Pappa et al. 2020; Salari et al. 2020; Salazar de Pablo et al. 2020) may have contributed to higher SA (Edge et al. 2022). In our study, 6.3% of the sample had mental health related SA within the first 2 years of the COVID‐19 pandemic. This can be compared to the prevalence of long‐term SA related to mental health problems among healthcare workers in Sweden during the years 2016–2018. The rates were 3.5%, 2.8%, and 1.4% for assistant nurses, nurses, and physicians respectively (AFA Försäkring 2020).

Among the study participants with SA, most were from known risk groups: they were more likely to be younger, assistant nurses, have a history of seeking treatment for mental health issues, and previous SA. Younger nurses, possibly due to their lack of experience making them less resilient to stressors, and assistant nurses, who work closely with patients but have little impact on decision‐making, were more susceptible to moral stress during the COVID‐19 pandemic (Gustavsson et al. 2023). Participants from these groups are therefore likely more vulnerable to the increased demands following a crisis (de Vries et al. 2018; Svedberg et al. 2018). Interestingly, there was no difference in the number of frontline workers between the SA groups. Although frontline work was related to many stressors during the COVID‐19 pandemic (Newman et al. 2021), frontline workers also felt important and needed (Zhang et al. 2021) and may therefore have been able to manage with higher workload without an increase in symptoms (Curtin et al. 2022).

### 5.1 Limitations

One major limitation of this study is that we did not differentiate between survey data collected at different times during the COVID‐19 pandemic in the analysis. This may affect variation in measured symptoms of psychological stress. However, individual trajectories among healthcare workers in Sweden with high levels of both burnout and depression seem to be relatively stable throughout the pandemic (Appelbom et al. 2024), we assumed that symptoms measured at different times could be included in the same analysis.

The use of ad‐hoc created single items when measuring sleep disturbances, detachment, and recovery may have affected the accuracy of the measurement of those symptoms. To limit this risk, these items were formulated based on existing questionnaires and pilot tested within the target population. However, to gain more reliable insights into the effects of sleep disturbances, detachment, and recovery on SA, instruments with known psychometric properties such as the detachment scale of the Recovery Experience Questionnaire (Sonnentag and Fritz 2007) are recommended for future studies.

When measuring SA related to mental health problems during the COVID‐19 pandemic, we used the number of net days during the total period, and we did not differentiate between episodes. Therefore, the measures < 90 SA days and ≥ 90 SA days are not equivalent to short‐ or long‐term SA (within the same episode); they simply measure more or fewer days of SA in total during the pandemic. Also, only net days of SA that occurred between the start of the COVID‐19 pandemic and the collection of survey responses were included in the analysis. Therefore, only the association between symptoms of psychological stress reactions and SA related to mental health problems can be established, and no causal conclusions can be drawn based on these study results.

## 6 Conclusions

The persistent crisis of the COVID‐19 pandemic, with repeated pandemic waves, brought new challenges in how to protect the health of healthcare workers long‐term (Gohar et al. 2020). The findings of this study may therefore help healthcare organizations prepare for future crises (WHO 2022a) by increasing their understanding of how symptoms of psychological stress unfold and relate to SA among their staff during a persistent crisis. Although 28% of participants belonged to the profile with high symptoms, only 6% were on SA during the pandemic. These results indicate that high symptoms of psychological stress during a crisis do not necessarily lead to serious mental health problems requiring SA. Further, this study provides insights into how various symptoms of psychological stress interrelate during a crisis. The results indicate that there are no specific symptoms of psychological stress that are particularly important to be conscious of among healthcare workers. Instead, similar to other contexts, symptoms of psychological stress are interrelated also during a crisis (Lindsäter et al. 2023). Because mental health related SA is highly contextual and driven not only by the experience of symptoms, future research should focus on how individual, social, and organizational factors may also contribute to mental health related SA among healthcare workers during a crisis.

## Author Contributions

**Sophia Appelbom:** conceptualization, data curation, formal analysis, investigation, methodology, project administration, visualization, writing – original draft, and writing – review and editing. **Anna Finnes:** conceptualization, data curation, investigation, methodology, supervision, and writing – review and editing. **Rikard K. Wicksell:** conceptualization, supervision, and writing – review and editing. **Aleksandra Bujacz:** conceptualization, funding acquisition, investigation, methodology, supervision, and writing – review and editing.

## Ethics Statement

The study was approved by the Swedish Ethical Review authority: Dnr 2020‐01795 with amendments (2020‐03495, 2020‐04959, 2020‐06602, and 2022‐01546‐02).

## Conflicts of Interest

The authors declare no conflicts of interest.

## Supporting information


Data S1:

## Data Availability

Raw data will not be shared due to the constraints in the information included in the written consent.
