# Supplementary material for: Symptoms of Psychological Stress and Sickness Absence Among Healthcare Workers During a Persistent Crisis
Source: Scand J Psychol. 2025 May 26;66(6):871–81. doi: 10.1111/sjop.13127 (PMC12611405; doi:10.1111/sjop.13127)
Supplement: Supplementary file 1 — Data S1: [file SJOP-66-871-s001.docx]

# Supplementary Materials

[Recruitment and reasons for non-responses 2](#_Toc190597559)

[Description of sub-cohorts 3](#_Toc190597560)

[R code 5](#_Toc190597561)

## Recruitment and reasons for non-responses

Data were retrieved from a larger research project with a total of 6889 participants contacted. Out of these, 1431 consented to the extraction of register data on sickness absence and where therefore eligible for the present study.

To be included in the analytic sample, participants needed to have scores on burnout (smbq), depressive symptoms (phq-2), anxiety (gad-7), symptoms of ptsd (pcl-5), sleep disturbance, lack of detachment, and lack of recovery (see Figure S1). A total of 14 participants dropped out from the entire survey after having provided written consent, and 71 participants answered parts of the survey but not the seven psychological stress symptoms variables. An additional 101 participants had partial response on the inclusion variables. Out of these, 13 participants belonged to a sub-cohort that did not receive the lack of detachment and lack of recovery items, and 54 participants belonged to a sub-cohort where burnout and anxiety scales were provided in a later survey. A total of 30 participants in this sub-cohort had responses only from either the first or second survey and thus did not have a complete response across all variables. For the remaining 4 participants with partial response on the inclusion variables, there are no methodological reasons for their non-responses.

**Figure S1**

*
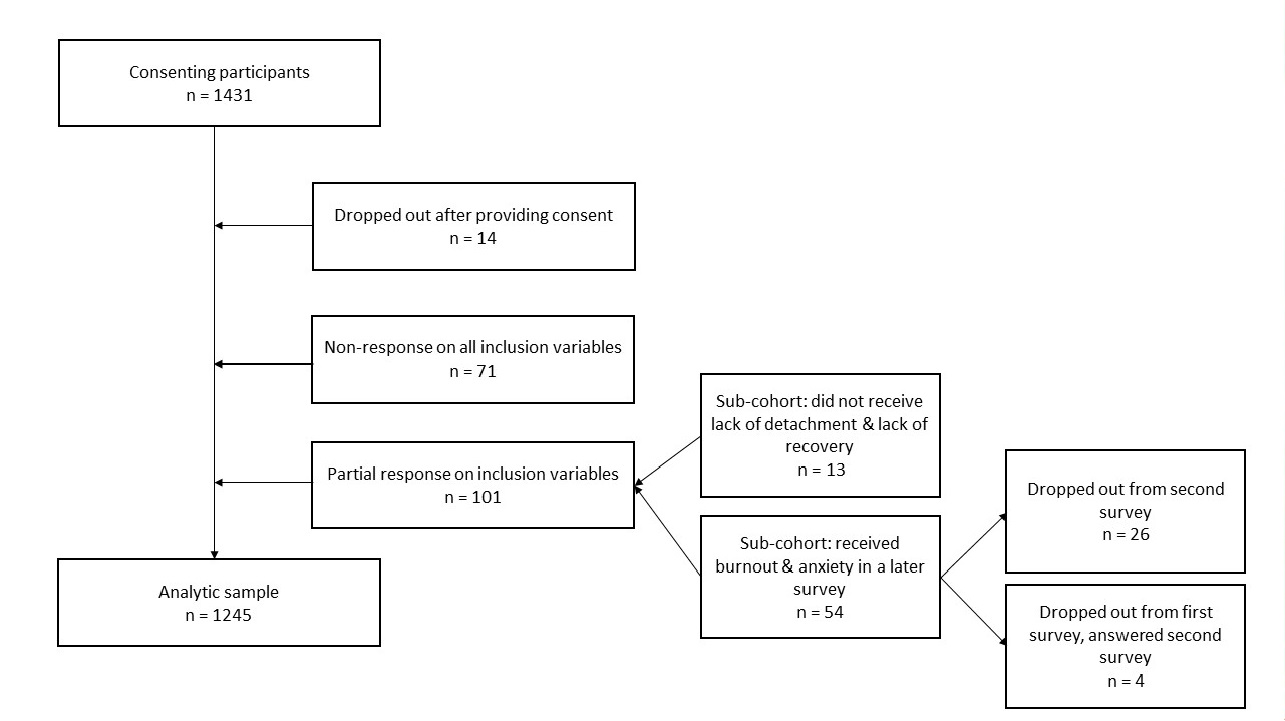
Flowchart of the inclusion process*

## Description of sub-cohorts

The study participants belonged to five different sub-cohorts participating either in a longitudinal survey with a baseline and four follow-ups (3 cohorts, *n* = 596), or a mobile daily stress intervention with survey baseline, pre- and post-follow-ups (2 cohorts, *n* = 835). At what time during the Covid-19 pandemic variables used in the present study were measured varies depending on which sub-cohort participants belonged to.

In the longitudinal survey, two sub-cohorts had their first survey in May and June 2020. In this first survey, measurements of PHQ-2, PTSD, Sleep disturbance, lack of detachment, and lack of recovery were included. In the second survey, SMBQ and GAD were included. Therefore, for participants from these two groups, depressive symptoms, symptoms of PTSD, sleep disturbance, lack of detachment and lack of recovery were measured in May or June 2020, while burnout and anxiety symptoms were measured in September 2020.

For the third sub-cohort from the longitudinal survey, the first survey was distributed in September 2020, and it included all variables. Therefore, for this sub-cohort all symptoms and recovery variables were measured at the same time, in September 2020.

In the mobile study, participants received a month-long intervention where they received tips on how to handle stress at work and logged how they were feeling every day in a mobile application. Before the start of the daily intervention, participants received baseline and pre-intervention surveys with a more extensive set of questions measuring their work environment, recovery patterns and psychological stress symptoms.

For sub-cohort 1 from the mobile study, all symptoms of psychological stress reactions and recovery from work were measured in a baseline survey distributed in December 2020. Sub-cohort 2 entered the mobile study when the pre-intervention survey was distributed. In that survey, measurements of sleep disturbance, detachment and recovery activities were not included. For this group of participants, measurements on sleep disturbance, lack of detachment and lack of recovery were included from the daily measurements in the mobile app intervention. In the intervention, questions were randomized across several days and each measure of a psychological stress reaction or recovery from work did not appear every day. Therefore, for each participant, we included the first day of ratings on the three variables of interest (sleep disturbance, lack of detachment, and lack of recovery).

For more information on sub-cohorts and an overview of when variables were measured, see Figure S2.

**Figure S2**


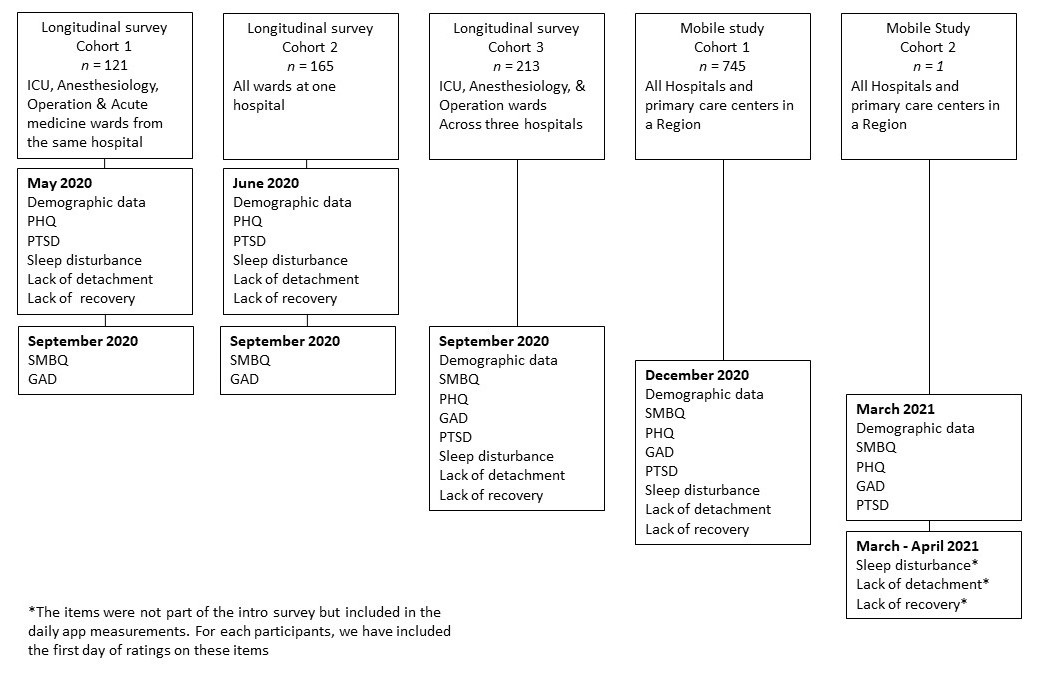
*Flowchart of data collection in different sub-cohorts.*

## R code

library(tidyverse)
library(tidySEM)
library(rcompanion)
library(FSA)

########LPA using tidySEM######################

Indicators = SA_total %>%

select(detach, leisure, sleepq, phq_index:gad_index)

#remove all cases that has some missing data

test_indicators = na.omit(Indicators)

#create centered indicators

center_variable <- function(x) {

if (is.numeric(x)) {

x - mean(x)

} else {

x

}

}

centered_indicators <- as.data.frame(lapply(test_indicators, center_variable))

#Step 1 finding profiles---------------------------------

NYres_cen <- mx_profiles(data = centered_indicators, classes = 1:8, variances = c("varying"), expand_grid = TRUE)

saveRDS(NYres_cen, "NYres_cen_gmm.RData")

NYres_cen <- readRDS("NYres_cen_gmm.RData")

fit_Nycen <- table_fit(NYres_cen)

plot(NYres_cen) + theme(axis.text.x = element_text(angle = 90, vjust = 0.5, hjust = 1))

res_Nyc_bic <- NYres_cen[["free var 4"]]

cpNy <- class_prob(res_Nyc_bic)

results_Nyc <- table_results(res_Nyc_bic, columns = c("label", "est", "std_est"))

results_Nyc

BLRT_Nycen = BLRT(NYres_cen, replications = 100)

plot_profiles(res_Nyc_bic, add_line = TRUE, rawdata = FALSE)

#---------size of profiles

res_NYcen_size = class_prob(res_Nyc_bic, type = "sum.mostlikely")

res_NYcen_size

#---------avarage posterior probabilities

class_prob(res_Nyc_bic, type = "avg.mostlikely")

########Group comparisons#############

# CHI-2 ###

library(rcompanion)

#prev_SA

chisq.test(table(test_predictors$lengthSA, test_predictors$prev_SA))

pairwiseNominalIndependence(table(test_predictors$lengthSA, test_predictors$prev_SA))

#post-hoc with st.residuals

chisq.test(table(test_predictors$prev_SA, test_predictors$lengthSA))$stdres

#notF_tot

chisq.test(table(test_predictors$lengthSA, test_predictors$notF_tot))

pairwiseNominalIndependence(table(test_predictors$lengthSA, test_predictors$notF_tot))

chisq.test(table(test_predictors$lengthSA, test_predictors$notF_tot))$stdres

#prev

chisq.test(table(test_predictors$lengthSA, test_predictors$prev))

pairwiseNominalIndependence(table(test_predictors$lengthSA, test_predictors$prev))

chisq.test(table(test_predictors$prev, test_predictors$lengthSA))$stdres #posthoc with st. residuals

#change

chisq.test(table(test_predictors$lengthSA, test_predictors$change))

#dfrontline

chisq.test(table(test_predictors$lengthSA, test_predictors$dfrontline))

#tenure

chisq.test(table(test_predictors$lengthSA, test_predictors$tenure))

#Children

chisq.test(table(test_predictors$lengthSA, test_predictors$Children))

#occupation

chisq.test(table(test_predictors$lengthSA, test_predictors$occupation))

pairwiseNominalIndependence(table(test_predictors$lengthSA, test_predictors$occupation))

pairwiseNominalIndependence(table(test_predictors$occupation, test_predictors$lengthSA))

chisq.test(table(test_predictors$occupation, test_predictors$lengthSA))$stdres #posthoc with std. residuals

#gender

test_predictors = test_predictors %>%

mutate(dgender = case_when(

gender == "0" ~ "0",

gender == "1" ~ "1",

gender == "9999" ~ NA

))

table(test_predictors$dgender, test_predictors$lengthSA)

chisq.test(table(test_predictors$lengthSA, test_predictors$dgender))

#Kruskal-wallis test

kruskal.test(detach ~ lengthSA, data = Xtest_predictors)

kruskal.test(leisure ~ lengthSA, data = Xtest_predictors)

kruskal.test(sleepq ~ lengthSA, data = Xtest_predictors)

kruskal.test(phq_index ~ lengthSA, data = Xtest_predictors)

kruskal.test(ptsd_index ~ lengthSA, data = Xtest_predictors)

kruskal.test(gad_index ~ lengthSA, data = Xtest_predictors)

kruskal.test(smbq_index ~ lengthSA, data = Xtest_predictors)

kruskal.test(age ~ lengthSA, data = test_predictors)

#adding effect sizes

library(rcompanion)

epsilonSquared(x = Xtest_predictors$detach,

g = Xtest_predictors$lengthSA)

epsilonSquared(x = Xtest_predictors$leisure,

g = Xtest_predictors$lengthSA)

epsilonSquared(x = Xtest_predictors$sleepq,

g = Xtest_predictors$lengthSA)

epsilonSquared(x = Xtest_predictors$phq_index,

g = Xtest_predictors$lengthSA)

epsilonSquared(x = Xtest_predictors$ptsd_index,

g = Xtest_predictors$lengthSA)

epsilonSquared(x = Xtest_predictors$gad_index,

g = Xtest_predictors$lengthSA)

epsilonSquared(x = Xtest_predictors$smbq_index,

g = Xtest_predictors$lengthSA)

#posthoc:

library(FSA)

dunnTest(leisure ~ lengthSA, data = Xtest_predictors, method="bonferroni")

dunnTest(phq_index ~ lengthSA, data = Xtest_predictors, method="bonferroni")

dunnTest(ptsd_index ~ lengthSA, data = Xtest_predictors, method="bonferroni")

dunnTest(gad_index ~ lengthSA, data = Xtest_predictors, method="bonferroni")

dunnTest(smbq_index ~ lengthSA, data = Xtest_predictors, method="bonferroni")

dunnTest(age ~ as.factor(lengthSA), data = test_predictors, method="bonferroni")

# Centered symptoms-------

center_variable <- function(x) {

if (is.numeric(x)) {

x - mean(x)

} else {

x

}

}

centered_symptoms <- as.data.frame(lapply(x, center_variable))

center_variable <- function(x) {

if (is.numeric(x)) {

x - mean(x)

} else {

x

}

}

vars_to_center <- c("detach", "leisure", "sleepq", "gad_index", "phq_index", "ptsd_index", "smbq_index")

centered_test_predictors <- test_predictors

centered_test_predictors[vars_to_center] <- lapply(test_predictors[vars_to_center], center_variable)

#dataframe with symptom means, divided by SA level

centered_mean_symptoms = centered_test_predictors %>%

group_by(lengthSA) %>%

summarize(

c_mean_detach = mean(detach),

c_mean_leisure = mean(leisure),

c_mean_sleep = mean(sleepq),

c_mean_phq = mean(phq_index),

c_mean_ptsd =mean(ptsd_index),

c_mean_smbq = mean(smbq_index),

c_mean_gad = mean(gad_index)

)
